# 5-Fluoro-1,3-dihydro-2,1-benzoxaborol-1-ol

**DOI:** 10.1107/S1600536811001632

**Published:** 2011-01-15

**Authors:** Izabela D. Madura, Agnieszka Adamczyk-Woźniak, Michał Jakubczyk, Andrzej Sporzyński

**Affiliations:** a Warsaw University of Technology, Faculty of Chemistry, Noakowskiego 3, 00-664 Warszawa, Poland

## Abstract

In the crystal structure of the title compound, C_7_H_6_BFO_2_, a broad-spectrum anti­fungal drug (AN2690), the planar [maximum deviation 0.035 (1) Å] mol­ecules form centrosymmetric *R*_2_^2^(8) dimers *via* strong O—H⋯O hydrogen bonds. The dimers are arranged into layers by weak inter­molecular C—H⋯O and C—H⋯F hydrogen bonds. The symmetry of this two-dimensional supra­molecular assembly can be described by the layer group *p* 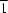 and topologically classified as a simple uninodal four-connected two-dimensional network of a (4.4.4.4.6.6) topology. Further weak C—H⋯O inter­actions build up the three-dimensional structure.

## Related literature

For the review of the synthesis, properties and applications of benzoxaboroles, see: Adamczyk-Woźniak *et al.* (2009 ▶). For the biological activity of the title compound, see: Baker *et al.* (2005 ▶, 2006 ▶); Hui *et al.* (2007 ▶); Rock *et al.* (2007 ▶). For the synthesis see: Baker *et al.* (2006 ▶), Gunasekera *et al.* (2007 ▶). For related structures, see: Adamczyk-Woźniak *et al.* (2010 ▶); Tan *et al.* (2001 ▶); Yamamoto *et al.* (2005 ▶); Zhdankin *et al.* (1999 ▶). For hydrogen-bond graph-set descriptors and layer symmetry groups, see: Etter (1990 ▶) and Inter­national Tables for Crystallography (2006 ▶), respectively.
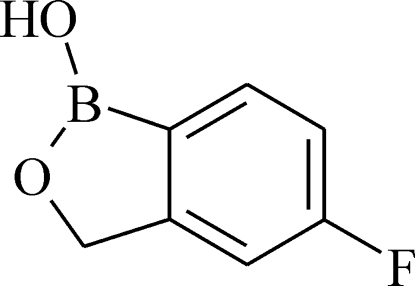


## Experimental

### 

#### Crystal data

C_7_H_6_BFO_2_
*M* *_r_* = 151.93
Triclinic, 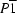

*a* = 3.8799 (3) Å
*b* = 6.3077 (5) Å
*c* = 14.0735 (12) Å
α = 98.068 (7)°
β = 91.564 (7)°
γ = 100.473 (7)°
*V* = 334.84 (5) Å^3^
*Z* = 2
Cu *K*α radiation
μ = 1.06 mm^−1^
*T* = 100 K
0.60 × 0.35 × 0.20 mm

#### Data collection

Oxford Diffraction Gemini A Ultra diffractometer
Absorption correction: multi-scan (*CrysAlis PRO*; Oxford Diffraction, 2006 ▶) *T*_min_ = 0.731, *T*_max_ = 1.000
3451 measured reflections
1193 independent reflections
1147 reflections with *I* > 2σ(*I*)
*R*_int_ = 0.016

#### Refinement

*R*[*F* ^2^ > 2σ(*F* ^2^)] = 0.032
*wR*(*F* ^2^) = 0.088
*S* = 1.07
1193 reflections
105 parameters
H atoms treated by a mixture of independent and constrained refinement
Δρ_max_ = 0.33 e Å^−3^
Δρ_min_ = −0.18 e Å^−3^

### 

Data collection: *CrysAlis PRO* (Oxford Diffraction, 2006 ▶); cell refinement: *CrysAlis PRO*; data reduction: *CrysAlis PRO*; program(s) used to solve structure: *SHELXS97* (Sheldrick, 2008 ▶); program(s) used to refine structure: *SHELXL97* (Sheldrick, 2008 ▶); molecular graphics: *ORTEPIII* (Burnett & Johnson, 1996 ▶); software used to prepare material for publication: *OLEX2* (Dolomanov *et al.*, 2009 ▶), *PLATON* (Spek, 2009 ▶) and *publCIF* (Westrip, 2010 ▶).

## Supplementary Material

Crystal structure: contains datablocks . DOI: 10.1107/S1600536811001632/fj2381sup1.cif

Structure factors: contains datablocks I. DOI: 10.1107/S1600536811001632/fj2381Isup2.hkl

Additional supplementary materials:  crystallographic information; 3D view; checkCIF report

## Figures and Tables

**Table 1:** Hydrogen-bond geometry (Å, °)

*D*—H⋯*A*	*D*—H	H⋯*A*	*D*⋯*A*	*D*—H⋯*A*
O2—H2⋯O1^i^	0.83 (2)	1.93 (2)	2.7614 (13)	175 (2)
C7—H7*B*⋯O2^ii^	0.99	2.55	3.5325 (15)	172
C5—H5⋯F1^iii^	0.95	2.58	3.4779 (14)	157
C7—H7*A*⋯O2^iv^	0.99	2.66	3.2172 (14)	116
C3—H3⋯O2^iv^	0.95	2.70	3.4276 (14)	134

Symmetry codes: (i) 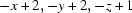; (ii) 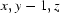; (iii) 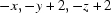; (iv) 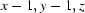.

**Fig. 1.**
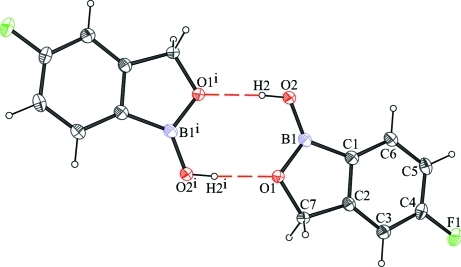
ORTEP plot of the hydrogen bonded dimer of (I), showing the atom-labelling scheme. Displacement ellipsoids are drawn at the 50% probability level.

**Fig. 2.**
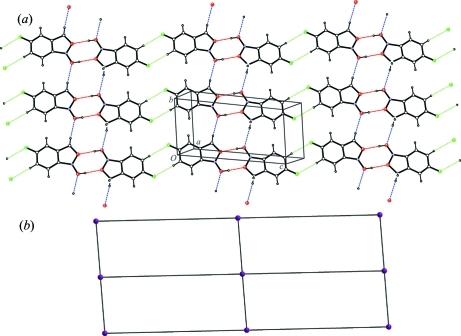
Projection on (1 0 2) plane showing layers of molecules linked by O—H···O (dashed lines), C—H···O and C—H···F (dotted lines) H-bonds.

**Fig. 3.**
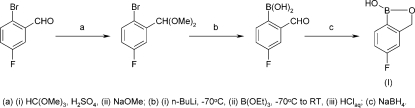
Synthesis of 5-fluoro-1,3-dihydro-1-hydroxy-2,1-benzoxaborole (I).

## supplementary crystallographic information

### Experimental

5-Fluoro-1,3-dihydro-1-hydroxy-2,1-benzoxaborole (I) was synthesized according to Fig. 3.

2-Bromo-5-fluorobenzaldehyde was purchased from Sigma-Aldrich and used as received. 2-Bromo-5-fluorobenzaldehyde (5.00 g, 0.025 mol) and 2.69 g (0.025 mol) of trimethoxymethane was dissolved in 100 ml of methanol in a two-necked vessel. 0.4 ml of concentrated H_2_SO_4_ was added. The solution was refluxed for one hour and left to cool down. Then the solution was brought to pH≈11 with a concentrated solution of NaOMe in methanol. The reaction mixture was distilled under vacuum to give 5.90 g of 1-Bromo-2-(dimethoxymethyl)-4-fluorobenzene as a colorless liquid (yield 96%; ^1^H NMR (CDCl_3_, 400 MHz): 7.49 (dd, 1H), 7.34 (dd, 1H), 6.91 (td, 1H), 5.49 (s, 1H), 3.37 (s, 6H) p.p.m.). The product was dissolved in 100 ml of dry Et_2_O in a three-necked vessel under argon flow. The solution was cooled down to -78°C using dry ice/acetone bath. n-Butyllithium in hexane (2.5 *M*, 11 ml) was added dropwise to keep the temperature under -70°C. The solution was stirred for one hour, then 3.80 g (0.026 mol, 4.4 ml) of triethyl borate was added slowly, keeping the temperature under -70°C. The dry ice/acetone bath was removed and the solution was stirred for one hour. The solution was brought to pH≈3 with 3 *M* aq. HCl. The aqueous layer was separated and extracted with Et_2_O (2 × 100 ml). The organic layers were combined and the solvent was partially removed under vacuum. The remaining thick solution was dissolved in hot water. Yellowish crystals of 4-fluoro-2-formylphenylboronic acid were filtered after a few hours. Recrystallization from water gave 1.79 g of the product (yield 49%; ^1^H NMR (CDCl_3_, 400 MHz): 9.89 (s, 1H), 8.31 (dd, 1H), 7.62 (dd, 1H), 7.40 (td, 1H) p.p.m.). The product (1.79 g, 0.011 mol) was dissolved in 100 ml of methanol in a one-necked vessel. 0.44 g (0.012 mol) of NaBH_4_ was added in small portions. The solution was mixed for 12 h. Another portion of 0.22 g of NaBH_4_ was added and the solution was mixed for 3 days. The solvent was removed under vacuum. The crude product was dissolved in water. Crystallization gave 0.82 g of 5-Fluoro-1,3-dihydro-1-hydroxy-2,1-benzoxaborole (I) as yellowish crystals (yield 51%; ^1^H NMR (CDCl_3_, 400 MHz): 7.72 (dd, 1H), 7.06 (m, 2H), 5.08 (s, 2H) p.p.m.; ^19^F NMR (CDCl_3_, 376.3 MHz): -113.51 (*q*) p.p.m.; ^11^B NMR ((CdD_3_)_2_CO, 64.1 MHz): 32.0 p.p.m.; m.p. 135–136°C).

### Refinement

H2 atom bonded to O2 atom was located in a difference map and freely refined. Other H atoms were positioned geometrically and refined using a riding model with C—H = 0.95–0.99 Å and with *U*_iso_(H) = 1.2 times *U*_eq_(C).

### Crystal data

**Table:**

C_7_H_6_BFO_2_	*Z* = 2
*M**_r_* = 151.93	*F*(000) = 156
Triclinic, *P*1	*D*_x_ = 1.507 Mg m^−^^3^
Hall symbol: -P 1	Melting point: 408 K
*a* = 3.8799 (3) Å	Cu *K*α radiation, λ = 1.5418 Å
*b* = 6.3077 (5) Å	Cell parameters from 3116 reflections
*c* = 14.0735 (12) Å	θ = 3.2–67.1°
α = 98.068 (7)°	µ = 1.06 mm^−^^1^
β = 91.564 (7)°	*T* = 100 K
γ = 100.473 (7)°	Prism, light yellow
*V* = 334.84 (5) Å^3^	0.60 × 0.35 × 0.20 mm

### Data collection

**Table:**

Oxford Diffraction Gemini A Ultra diffractometer	1193 independent reflections
Radiation source: Enhance Ultra (Cu) X-ray Source	1147 reflections with *I* > 2σ(*I*)
mirror	*R*_int_ = 0.016
Detector resolution: 10.3347 pixels mm^-1^	θ_max_ = 67.1°, θ_min_ = 3.2°
ω scans	*h* = −4→4
Absorption correction: multi-scan (*CrysAlis PRO*; Oxford Diffraction, 2006)	*k* = −7→7
*T*_min_ = 0.731, *T*_max_ = 1.000	*l* = −16→14
3451 measured reflections	

### Refinement

**Table:**

Refinement on *F*^2^	Secondary atom site location: difference Fourier map
Least-squares matrix: full	Hydrogen site location: inferred from neighbouring sites
*R*[*F*^2^ > 2σ(*F*^2^)] = 0.032	H atoms treated by a mixture of independent and constrained refinement
*wR*(*F*^2^) = 0.088	*w* = 1/[σ^2^(*F*_o_^2^) + (0.0511*P*)^2^ + 0.1152*P*] where *P* = (*F*_o_^2^ + 2*F*_c_^2^)/3
*S* = 1.07	(Δ/σ)_max_ < 0.001
1193 reflections	Δρ_max_ = 0.33 e Å^−^^3^
105 parameters	Δρ_min_ = −0.18 e Å^−^^3^
0 restraints	Extinction correction: *SHELXL97* (Sheldrick, 2008), Fc^*^=kFc[1+0.001xFc^2^λ^3^/sin(2θ)]^-1/4^
Primary atom site location: structure-invariant direct methods	Extinction coefficient: 0.046 (5)

### Special details

**Table:**

Experimental. Empirical absorption correction using spherical harmonics, implemented in SCALE3 ABSPACK scaling algorithm. (Oxford Diffraction, 2006)
Geometry. All e.s.d.'s (except the e.s.d. in the dihedral angle between two l.s. planes) are estimated using the full covariance matrix. The cell e.s.d.'s are taken into account individually in the estimation of e.s.d.'s in distances, angles and torsion angles; correlations between e.s.d.'s in cell parameters are only used when they are defined by crystal symmetry. An approximate (isotropic) treatment of cell e.s.d.'s is used for estimating e.s.d.'s involving l.s. planes.
Refinement. Refinement of *F*^2^ against ALL reflections. The weighted *R*-factor *wR* and goodness of fit *S* are based on *F*^2^, conventional *R*-factors *R* are based on *F*, with *F* set to zero for negative *F*^2^. The threshold expression of *F*^2^ > σ(*F*^2^) is used only for calculating *R*-factors(gt) *etc*. and is not relevant to the choice of reflections for refinement.

### Fractional atomic coordinates and isotropic or equivalent isotropic displacement parameters (Å^2^)

**Table:**

	*x*	*y*	*z*	*U*_iso_*/*U*_eq_	
O2	0.9636 (2)	1.22613 (14)	0.60638 (7)	0.0189 (3)	
F1	0.0385 (2)	0.70856 (13)	0.93059 (5)	0.0282 (3)	
O1	0.7364 (2)	0.84381 (13)	0.56821 (6)	0.0171 (3)	
C3	0.2566 (3)	0.6725 (2)	0.77652 (9)	0.0185 (3)	
H3	0.1624	0.5207	0.7660	0.022*	
C4	0.2168 (3)	0.8036 (2)	0.86129 (9)	0.0200 (3)	
C2	0.4422 (3)	0.7757 (2)	0.70750 (9)	0.0162 (3)	
C1	0.5825 (3)	0.9983 (2)	0.72234 (9)	0.0167 (3)	
C5	0.3485 (3)	1.0251 (2)	0.87950 (9)	0.0207 (3)	
H5	0.3121	1.1083	0.9388	0.025*	
C6	0.5346 (3)	1.1237 (2)	0.80968 (9)	0.0186 (3)	
H6	0.6291	1.2754	0.8210	0.022*	
B1	0.7783 (3)	1.0404 (2)	0.63011 (10)	0.0164 (3)	
C7	0.5248 (3)	0.6711 (2)	0.61026 (9)	0.0170 (3)	
H7B	0.6562	0.5526	0.6164	0.020*	
H7A	0.3064	0.6096	0.5701	0.020*	
H2	1.061 (5)	1.212 (3)	0.5545 (15)	0.040 (5)*	

### Atomic displacement parameters (Å^2^)

**Table:**

	*U*^11^	*U*^22^	*U*^33^	*U*^12^	*U*^13^	*U*^23^
O2	0.0245 (5)	0.0147 (5)	0.0169 (5)	0.0025 (4)	0.0053 (4)	0.0011 (3)
F1	0.0329 (5)	0.0323 (5)	0.0196 (4)	0.0035 (4)	0.0106 (3)	0.0065 (3)
O1	0.0208 (5)	0.0145 (5)	0.0154 (5)	0.0019 (3)	0.0046 (3)	0.0017 (3)
C3	0.0178 (6)	0.0188 (6)	0.0193 (7)	0.0037 (5)	0.0009 (5)	0.0033 (5)
C4	0.0181 (6)	0.0273 (7)	0.0160 (6)	0.0052 (5)	0.0038 (5)	0.0061 (5)
C2	0.0149 (6)	0.0174 (6)	0.0168 (6)	0.0055 (5)	−0.0004 (4)	0.0014 (5)
C1	0.0154 (6)	0.0174 (6)	0.0178 (7)	0.0051 (5)	−0.0011 (5)	0.0021 (5)
C5	0.0212 (6)	0.0256 (7)	0.0155 (6)	0.0083 (5)	0.0008 (5)	−0.0016 (5)
C6	0.0187 (6)	0.0179 (6)	0.0188 (6)	0.0049 (5)	−0.0001 (5)	−0.0007 (5)
B1	0.0162 (6)	0.0162 (7)	0.0172 (7)	0.0055 (5)	−0.0011 (5)	0.0009 (5)
C7	0.0197 (6)	0.0135 (6)	0.0174 (6)	0.0017 (5)	0.0036 (5)	0.0021 (5)

### Geometric parameters (Å, °)

**Table:**

O2—B1	1.3483 (18)	C2—C1	1.3948 (18)
O2—H2	0.83 (2)	C2—C7	1.5025 (17)
F1—C4	1.3562 (15)	C1—C6	1.4013 (17)
O1—B1	1.3922 (17)	C1—B1	1.5522 (18)
O1—C7	1.4471 (15)	C5—H5	0.9500
C3—H3	0.9500	C5—C6	1.3856 (18)
C3—C4	1.3822 (19)	C6—H6	0.9500
C3—C2	1.3897 (18)	C7—H7B	0.9900
C4—C5	1.3829 (19)	C7—H7A	0.9900
			
O2—B1—O1	121.51 (12)	C2—C3—H3	121.9
O2—B1—C1	130.25 (12)	C2—C1—C6	119.16 (12)
F1—C4—C3	117.85 (12)	C2—C1—B1	104.93 (11)
F1—C4—C5	118.27 (12)	C2—C7—H7B	110.7
O1—B1—C1	108.24 (11)	C2—C7—H7A	110.7
O1—C7—C2	105.45 (9)	C1—C2—C7	110.88 (11)
O1—C7—H7B	110.7	C1—C6—H6	120.2
O1—C7—H7A	110.7	C5—C6—C1	119.66 (12)
C3—C4—C5	123.88 (12)	C5—C6—H6	120.2
C3—C2—C1	122.36 (12)	C6—C1—B1	135.86 (12)
C3—C2—C7	126.75 (11)	C6—C5—H5	120.6
C4—C3—H3	121.9	B1—O2—H2	115.3 (13)
C4—C3—C2	116.12 (12)	B1—O1—C7	110.46 (10)
C4—C5—H5	120.6	H7B—C7—H7A	108.8
C4—C5—C6	118.82 (12)		
			
F1—C4—C5—C6	179.28 (10)	C2—C1—B1—O2	−179.25 (12)
C3—C4—C5—C6	−0.58 (19)	C2—C1—B1—O1	0.71 (13)
C3—C2—C1—C6	−0.23 (17)	C1—C2—C7—O1	2.08 (13)
C3—C2—C1—B1	177.60 (11)	C6—C1—B1—O2	−2.0 (2)
C3—C2—C7—O1	−177.19 (11)	C6—C1—B1—O1	178.00 (12)
C4—C3—C2—C1	0.24 (18)	B1—O1—C7—C2	−1.57 (13)
C4—C3—C2—C7	179.42 (11)	B1—C1—C6—C5	−177.18 (12)
C4—C5—C6—C1	0.57 (18)	C7—O1—B1—O2	−179.45 (10)
C2—C3—C4—F1	−179.69 (9)	C7—O1—B1—C1	0.59 (13)
C2—C3—C4—C5	0.17 (19)	C7—C2—C1—C6	−179.53 (10)
C2—C1—C6—C5	−0.19 (17)	C7—C2—C1—B1	−1.70 (13)

### Hydrogen-bond geometry (Å, °)

**Table:**

*D*—H···*A*	*D*—H	H···*A*	*D*···*A*	*D*—H···*A*
O2—H2···O1^i^	0.83 (2)	1.93 (2)	2.7614 (13)	175 (2)
C7—H7B···O2^ii^	0.99	2.55	3.5325 (15)	172
C5—H5···F1^iii^	0.95	2.58	3.4779 (14)	157
C7—H7A···O2^iv^	0.99	2.66	3.2172 (14)	116
C3—H3···O2^iv^	0.95	2.70	3.4276 (14)	134

Symmetry codes: (i) −*x*+2, −*y*+2, −*z*+1; (ii) *x*, *y*−1, *z*; (iii) −*x*, −*y*+2, −*z*+2; (iv) *x*−1, *y*−1, *z*.
